# Azoturia1Read at the Thirty-sixth Annual Meeting of the American Veterinary Medical Association, New York, September, 1899.

**Published:** 1899-09

**Authors:** Richard P. Lyman

**Affiliations:** Hartford, Conn.


### AZOTURIA.[1]

By Richard P. Lyman, M.D.V.,
HARTFORD, CONN.

In presenting this paper here to-day I shall ask that you will overlook any shortcomings, for my investigations upon the subject of this essay are still far from being in a finished condition, therefore I am unable to present as complete a paper as I would wish. Indeed, my object in selecting this subject is very largely the hope that by it further thought and investigation may be brought to help elucidate the many perplexing questions presented to us by a disease which is known as azoturia—a term expressive of nothing further than the presence of nitrogenous matter in the urine, and giving us no knowledge why it should be there or what conditions accompany it. Williams named it azoturia because he thought he uniformly found uric acid to excess in the urine. He distinctly stated that (in the earlier editions, at any rate) there was no blood in the urine nor any of its constituents.

A relation of the causes and symptoms, so well known to you

[1] Read at the Thirty-sixth Annual Meeting of the American Veterinary Medical Association, New York, September, 1899.

all, may well be passed with a general outline. They are not entirely without complications, and vary in different cases. More especially we observe at the outset an irregular gait, manifesting its presence at varying periods after the commencement of exercise. This increases to lameness of more or less severity in one or both hind legs, and is accompanied with uneasiness and more or less profuse perspiration, in turn followed, more especially if the animal is urged to move, by inability to stand and possibly symptoms of severe colic, the pain varying in duration and rapidly followed by the ordinary signs of azoturia and muscular inability.

In the consideration of its pathology, which I approach with considerable hesitancy, let us begin by remembering the causes of azoturia. Here we find the principal primary factor is overfeeding during enforced idleness. But how can these conditions, so comparatively harmless in other animals, bring about azoturia? Animals are fed to bring about, first, a substitute for the tissues of the body that are continuously breaking down or needing growth ; second, to maintain a regulated heat-supply of the body; and, third, to furnish sufficient force and energy for work and proper functional activity. Thus it is evident that food should always be regulated by the kind and amount of work to be performed.

A horse whose feed has been so proportioned that a proper equilibrium has been established between the ingesta and egesta while regularly worked, suddenly forced into idleness without decrease of that ratio, has a large portion of nutrition, formerly used up by energy, functional activity, and the production of heat, that is not now required. What does nature do with this surplus? By answering this query I shall hope to present my theory of azoturia.

Physiology teaches that a function of the liver is to secrete and excrete, to arrest and render harmless the toxic substance arising within the alimentary canal by means of the bile, which is made not in the blood, but is secreted from the blood by the liver, and that this bile is, first, an aid to the emulsification of fats; second, to the absorption through the bowels of its digested contents ; and, third, that it has some antiseptic properties in controlling the evil influences of the toxins that may be formed within the intestines; this latter office can be experimentally demonstrated by the effects of bile upon the products of fermentation. The ultimate destination of this bile is to be in part discharged as excrementations and in part to be returned to the blood and again secreted by the liver.

From the peculiar manner in which the liver is supplied with blood it is extremely probable that this organ is excretory not only for such hydrocarbonaceous matters as may need expulsion from the blood, but that it serves also for the direct purification of the stream, which, arriving by the portal vein, has just gathered up various substances in its course through the digestive system—substances which may need to be expelled almost at once upon being absorbed. A further office of the liver is its glycogenic functions—a process within its cells for preparing material for the use of the body; this material passes from the hepatic into the general circulation, to be stored up in the muscular system of the animal economy in some form of lactic acid or compounds capable of forming urea by the metabolism of the muscles. Beyond this physiological action of the liver, nature at the same time attempts to overcome the excess of nutrition by the excretions from the bowels and kidneys. In addition to this, overfeeding and enforced idleness induce a diminished peristalsis, with consequent overloading of the bowel, which in turn, by hindering digestion, so increases fermentation in the mass and causes an abnormal production of intestinal toxins to be mixed with the general product as it is made ready for absorption by the lacteals, the office of which system is to take up the digestive elements of the food from the bowels and pour them into the thoracic duct, from whence they enter the general circulation. We therefore find, as has been shown, three distinct and important sources by which impurities induced in the etiology of azoturia may enter the blood, and may next consider the probable effects of these impurities upon the blood as a whole.

In solipeds the anatomical arrangement of the liver is such that its secretion of bile is not discharged from a reservoir, as in most species of animals, but is passed directly from that organ into the intestinal canal. It is, therefore, a matter of considerable importance that the liver shall always be free and able to act to the full extent; but the unfortunate fact is that one of the earlier effects of the general condition is upon the liver, whereby it is rendered unfit to perform anything like its free function; it soon becomes engorged and throws polluted blood through the system, which must be purified by some other organ or cause the death of the animal. Purification of the blood is variously accomplished by the bowels, the kidneys, the lungs, and, to some extent, the skin. Under the existing circumstances the bowels are not to be depended upon to render aid, and as a consequence by far the greater part of the work falls upon the kidneys. These organs are now severely taxed to remove from the general circulation and system the abnormal quantity of urea and toxin. There now exists a tired kidney straining its every effort to establish an equilibrium. It has repeatedly been shown that if at this period the animal is allowed to continue to be inactive for a sufficient length of time this equilibrium becomes established without any apparent resulting harm, for although cases of azoturia have been reported in the journals as originating in the stable, the exception is so rare that its explanation may well be left to take care of itself upon the general hypothesis. When, however, at the time short of this period the animal is again put to work, the muscular activity so induced generates increased heat, the influence of which upon the stored-up nitrogenous crystallizable substances which have been produced in excess during the period of rest is to produce an excess of muscular waste, or, in other words, the formation of compounds of urea in excess—an increasedly rapid change of arterial into venous blood, with a correspondingly relative increase of carbon dioxide in the system.

Of these sudden changes radiation regulates the production of heat, equalizing the body temperature; carbon dioxide is passed off, mostly by the lungs by stimulated respiration, and the urea, although somewhat removed by radiation, must mostly be disposed of by the excretory organs, namely, the bowels and kidneys. The former being sluggish in action, throw the work, as before stated, upon the already overworking, but struggling kidneys.

The kidneys are likened to a machine—they can stand but a limited amount of tension before they must give way. Blood pours into them, engorging them with poisonous substances which they cannot accommodate, and naturally they give way under the strain and present a condition of acute and generally severe hyperæmia. While the kidneys themselves are not primarily diseased it is exceedingly probable that the amount of toxic material introduced into the blood-stream is in many cases in excess of the ability of the kidneys to throw off.

Normal urine from the horse is of a yellowish-white color, somewhat cloudy. In azoturia it is of a dark brown or reddish-black color, due to a large amount of blood-hæmatin that has escaped during the acute hyperæmia. A chemical examination of this urine discloses the existence of a large amount of urea and uric acid; the presence of albumin and various other constituents of the blood, and due here mostly to the escape of blood constituents.

Its microscopical examination presents blood globules in various stages of decomposition, epithelium from the kidneys, with occasional blood and epithelium casts. Newly passed urine—before any decomposition has occurred—has normally an alkaline reaction; in the disease under consideration I find it faintly acid or neutral; it, however, rapidly becomes alkaline and ammoniacal from the decomposition of the urea into compounds of ammonia.

Having found a general derangement of the bowels, liver, and kidneys, likewise impaired circulatory fluid, we have by no means reached the end of our trouble, for there is still one prominent symptom that remains to be pathologically accounted for; it is that of the inability of the hind-quarters. If this is to be explained on a theory of simple nervous derangement due to the circulation of toxic blood, it is difficult to account for the fact that the following paralysis affects the hind extremities alone, or, perhaps, only one of them, and that the brain, while the circulatory and respiratory government remains in the comparatively perfect condition in which we find it in cases that have long lost the power to stand. If we make the attempt to explain the phenomena by a theory based upon the local innervation we find that the nerve-supply to the kidneys is from the renal plexus, which, in turn, is derived from the solar plexus, particularly its semi-lunar ganglion, that the renal plexus is indirectly connected with the splanchnic nerves, and also directly connected by nerves going direct without going first to the solar plexus. Upon a first consideration of this anatomical arrangement it would seem to be plausible to say that anything within the digestive organs that has a marked influence upon the splanchnic nerve-fibres might readily influence the action of the renal plexus, or *vice versa*, and that this might more particularly apply to those fibres and ganglionic centres lying in close proximity to the disabled kidneys, where there is now a congestion which causes a continued unusual pressure upon the nerves—this continuous excessive activity of the nerves would be finally abolished and paralysis result; but the fact is that the inability to stand precedes the occurrence of any change in the kidneys that is sufficient to bring about a paralysis which can clearly be explained upon this basis.

A more attractive theory for the production of a paralysis of the hinder parts, it seems to me, is to be found by considering that there is produced a reflex stimulation from the torpid bowels and liver which may travel backward along the splanchnic to the ganglion of the lumbar region, and so bring it about, even increasing its force as time and this unrelieved condition goes on. In further defence of this position a very proper appeal to analogy may be made by instancing a similar condition of paraplegia so often witnessed in dogs suffering from a similar impacted condition of the bowels. The accompanying condition of hardness and fulness of the gluteals, with possibly a more or less severe cramp of them, may perhaps be accounted for by remembering that a series of stimulations to a muscle, as from the impacted bowel in this instance, reflexly, as explained, may produce a clonic spasm in the muscle or muscles so stimulated. Further, we know that if overstimulation of a muscle or group of muscles be long continued, it or their power of responding to stimulus may be lost, and that there will then ensue a condition of more or less absolute paralysis, which, in its turn, if long continued will absolutely impair the nutrition of the parts, and so add to our embarrassment in our attempt to return them to their normal condition. Indeed, we occasionally see, following certain cases of azoturia otherwise perfectly recovered, this loss of power of nutrition in the affected muscles carried to an extent which produces waste of their bulk and a continuance of their inability, which lasts throughout the remainder of the animal's life.

It has been my aim thus far to demonstrate that azoturia is a condition which is brought about by an injudicious overtaxing of the digestive powers by the allowance of too much food, the nutritive value of which is far greater than can be taken care of by the amount of idleness to which the animal is of necessity compelled; that this is followed, as soon as exercise increases metabolism to a certain degree, by a condition of engorgement of the liver, with a consequent overloading of the blood with poisonous ptomaïns, to relieve which nature, under the existing torpid condition of the bowels, is obliged to rely almost entirely upon the kidneys; that these last, under the tremendous pressure thus suddenly thrown upon them, if unrelieved, break down, and the animal dies of toxæmia.

It seems to me that the pathological anatomy of animals dead of azoturia will fully substantiate this position. In those that have died without extended medical treatment or catharsis of the bowels I have universally found the bowels to be full and compact, with diminished watery secretions; the liver is always found congested, engorged, and ready to bleed freely upon section. The kidneys present a condition of severe congestion, and, like the liver, are soft and engorged with blood. In one case I discovered absolutely no urine present in the bladder or kidneys, and none had escaped for at least six hours before death, at which time I passed a catheter without results; the animal had been down two days without medical attendance. I have found the nerve-fibres passing from the lumbar region posteriorly, surrounded by a congested covering, especially in protracted cases.

In attempting to formulate a system of treatment for azoturia it will, of course, be understood that in this disorder, as in others of equal gravity, some cases must have a fatal termination, however thoroughly its pathology may be understood. There will be cases which cannot be made to yield to any form of treatment, the systemic disturbances being so severe that treatment is unproductive; however, it does appear to me that proper management can lower the percentage of mortality to a large degree. The basis of treatment must be directed to some system or method that will relieve the pathological changes as rapidly as possible.

To properly systematize a treatment a correct diagnosis is the first fundamental principle. In azoturia the diagnosis is by far less difficult than in a majority of the ailments affecting the horse; the history and examination of the urine will generally suffice to differentiate this from other troubles—those more likely to be confounded being cerebro-spinal meningitis, paraplegia from other causes, colics, and nail-pricks.

Upon reaching an undisputed diagnosis we must direct our treatment to both the symptoms and pathology. Observance of the former is requisite, owing to the liability of complicating symptoms and the exact stage in which our patient may be when first visited. As stated, many cases are, when first seen, suffering more or less colicky pains; this can be very much relieved and successfully overcome by a full-sized dose of cannabis indica, and this without any of the ill effects that I have seen follow the administration of hypodermatic injections of eserine. To be sure, the latter occasionally gives a quick relief to the overloaded bowels; but following its use there is almost invariably an increase of the general paralysis, which prolongs the case, so rendering the final cure less possible. The complicating symptoms having been attended to as the occasion demands, treatment must be directed to the disease itself, continually bearing in mind the pathology.

Knowing the torpid condition of the bowels and liver, it is advisable to administer some strong cholagogue, combined with a brisk cathartic; this can be accomplished by a one-quarter to one-half drachm of calomel with eight to nine drachms of aloes; a drench, shaken frequently during administration, acts more readily than when these are given in a bolus. Colchicum or podophyllin may be substituted for calomel for hepatic stimulants, and will not produce superpurgation as readily in combination with aloes. If by these measures purgation is established the bowels will be able to perform some of the work which must otherwise continue to fall upon the already overworked kidneys.

As a relief for the hyperæmic kidneys I find the frequent use of the citrate of lithium beneficial; this has less of the depressant effects so marked in most of the alkaline metals; it is a powerful solvent of uric acid, an efficacious diuretic, and will very readily render the urine alkaline. The paralytic symptoms can be treated by both external and by internal medication. Externally I would recommend a complete covering of the spine forward as far as the withers, with a mixture of euphorbia and mustard, well rubbed in, covered with a piece of paper, and allowed to remain on for about one-half to three-quarters of an hour. Upon its removal the parts should be warmly covered with dry blankets, or, better, if in a place where it can be successfully accomplished, a frequent changing of blankets that have been wrung out of boiling water. These blankets retain their heat longer if covered with dry woollen blankets. This method being pursued in close proximity to the kidneys may have some stimulating effects upon them which might seem out of place, but when accompanied with the treatment brings about sufficient benefit to the muscles and nerves to offset its harmful action upon them. In the latter stages electricity may be tried externally for muscular stimulation. My experience with it has not been followed with sufficient benefit to recommend it, and although I have employed it a number of times during the past three years, the only use I have given it this year was absolutely devoid of results.

For further medical treatment I begin the administration from the outset of the hypodermic injections of one-sixth of a grain of strychnine, repeated each two hours until the action of the drug is noticed or the animal shows signs of regaining its limbs. Therapeutics teaches us that strychnine is a violent poison in sufficient quantities, causing tonic or clonic convulsions of all the voluntary muscles. Light doses, on the other hand, produce correspondingly slight stimulation to these muscles, accompanied by periods of complete relaxation. Strychnine not only stimulates the motor nervous system, but it also increases the activity of the non-striated muscles; thus it aids in the rapidity of the action of the cathartic and cholagogue; medicinal doses strengthen the beat of the heart; strychnine thus acts as an excitor motor and stimulant to the nerves and muscles that are inactive in azoturia.

Medical treatment by no means constitutes the entire care of this disease. Mechanical treatment must also constitute an important adjunct. Paralysis of the bladder must necessitate a frequent removal of its contents. It has been the general practice among practitioners to ease the patient and prevent so-called bedsores by frequent turning from side to side. This is accomplished by rolling upon the back and over. My opinion is that this is a very unsafe procedure, upsetting the general system too suddenly, more especially the circulatory. My practice is to place slings upon all cases of azoturia as soon as they have reached a place for permanent treatment, and an attempt should be made about every three hours to place the patient upon its feet, thus giving it a chance to try its muscles, straighten its legs, and try its power of using its limbs. I do not allow the animal, even if it can, to stand, if so doing seems to cause pain. In letting them down from the slings I so arrange that the animal will lie upon the opposite side from which it had last reposed. This treatment has been carefully followed in seventeen cases since the beginning of the year, resulting in a loss of one animal—*i. e.*, about 94 per cent. of recoveries. Of the seventeen cases nine have been paralyzed to the extent of inability to stand, either with or without the aid of slings, complete recovery occurring in all but two. As before stated, one of the two died (after lying a week); the other, Case X., gradually regained her feet, but never freely recovered the use of her off hind-leg.

